# Closely linked *cis*-acting modifier of expansion of the CGG repeat in high risk *FMR1* haplotypes

**DOI:** 10.1002/humu.20600

**Published:** 2007-08-02

**Authors:** S Ennis, A Murray, G Brightwell, NE Morton, PA Jacobs

**Affiliations:** 1 Genetic Epidemiology Group, Human Genetics (MP808), Southampton General HospitalSouthampton, United Kingdom; 2 Peninsula Medical School, St. LukesExeter, United Kingdom; 3 AgResearchHamilton, New Zealand; 4 Wessex Regional Genetics Laboratory, Salisbury District HospitalSalisbury, Wiltshire, United Kingdom

**Keywords:** fragile X, *cis*-acting, expansion, modifier

## Abstract

In its expanded form, the fragile X triplet repeat at Xq27.3 gives rise to the most common form of inherited mental retardation, fragile X syndrome. This high population frequency persists despite strong selective pressure against mutation-bearing chromosomes. Males carrying the full mutation rarely reproduce and females heterozygous for the premutation allele are at risk of premature ovarian failure. Our diagnostic facility and previous research have provided a large databank of X chromosomes that have been tested for the FRAXA allele. Using this resource, we have conducted a detailed genetic association study of the FRAXA region to determine any *cis*-acting factors that predispose to expansion of the CGG triplet repeat. We have genotyped SNP variants across a 650-kb tract centered on FRAXA in a sample of 877 expanded and normal X chromosomes. These chromosomes were selected to be representative of the haplotypic diversity encountered in our population. We found expansion status to be strongly associated with a ∼50-kb region proximal to the fragile site. Subsequent detailed analyses of this region revealed no specific genetic determinants for the whole population. However, stratification of chromosomes by risk subgroups enabled us to identify a common SNP variant which cosegregates with the subset of D group haplotypes at highest risk of expansion (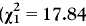, p=0.00002). We have verified that this SNP acts as a marker of repeat expansion in three independent samples. Hum Mutat 28(12), 1216–1224, 2007. © 2007 Wiley-Liss, Inc.

## INTRODUCTION

There are now more than 35 genes identified in which variable number tandem repeats cause disease [Pearson et al., 2005]. This mechanism of mutation was first described in the FMR1 gene (MIM# 309550; GenBank accession number L29074.1) in which expansion of a CGG repeat in the 5′ untranslated region was associated with the fragile X syndrome [Verkerk et al., 1991]. The expansion mutations which cause fragile X syndrome are over 200 repeats and cause methylation of the promoter and subsequent inactivation of the gene. In the general population the CGG repeat is polymorphic, with repeats ranging from six to 50 and modes at 20, 23, 30, and 40 in Caucasian populations. Repeats between 50 and 200 are usually unmethylated and are termed premutations. The expansion mutation in fragile X is inherited maternally and invariably originates from a premutation. Premutations are at risk of expanding to a full mutation dependent on the size of the repeat, with repeats over 100 at 100% risk. However, repeats as small as 59 have been shown to expand to a full mutation in a single transmission [Nolin et al., 2003].

The trinucleotide repeat in FMR1 is not composed entirely of CGGs, but is interspersed with AGGs. In normal alleles, AGGs are typically present at the 9th or 10th repeat in the tract from the 5′ end and again at the 19th or 20th repeat, with the majority of variation in repeat composition occurring at the 3′ end. In approximately 50% of premutations there are no AGG interruptions, with the majority of the remaining 50% having a single AGG at the 5′ position, i.e., the 9th/10th repeat. This suggests that loss of 3′ AGGs is a fundamental event during progression to a premutation. Whether loss of AGGs cause the expansion or is a result of the expansion is not known. The length of the longest uninterrupted CGG repeat is thought to be an important determinant for instability, but the number and position of AGGs may also influence stability [Crawford et al., 2000b; Eichler et al., 1996; Kunst et al., 1996; Murray et al., 1997; Mathews et al., 2001].

Mouse models have attempted to mimic the meiotic CGG repeat instability seen in humans. However, to date, instability has been limited even when up to 300kb of flanking human sequence is included in the transgene and there are no sex differences in expansion risk in mice [Bontekoe et al., 2001; Brouwer et al., 2007; Lavedan et al., 1998; Peier and Nelson, 2002]. Recently, expansions of about 140 repeats have been observed in mice with a 120–repeat transgene, but these were in the minority of transmissions (˜0.5%), in contrast to humans in which alleles of this size would invariably expand to a full mutation during female transmission [Entezam et al., 2007]. These data suggest that repetitive DNA alone is not intrinsically unstable. It is possible that differences in DNA replication or repair machinery between species account for the observed differences in instability; however, to date these have not been identified. An alternative explanation is that genetic background extending beyond the 300kb in the mouse model is an important determinant of instability.

Many studies in different ethnic groups have shown that full mutations are more likely to occur on certain microsatellite haplotype backgrounds. Haplotypes are also associated with repeat size and AGG interspersion pattern in normal alleles. These studies have led to the suggestion that there are at least three pathways for progression to a full mutation [Eichler et al., 1996; Macpherson et al., 1994]. In some Caucasian populations, between 15 and 25% of full mutation expansions occur on the 2–1–3 haplotype [Buyle et al., 1993; Chiurazzi et al., 1996; Crawford et al., 2000a; Larsen et al., 2000; Macpherson et al., 1994; Murray et al., 1997]. This haplotype is also associated with alleles with between 40 and 60 repeats (intermediate), which are often interspersed with three or more AGGs. It is suggested that these alleles accumulate CGGs gradually but remain relatively stable because they are highly interrupted. However, random loss of an AGG in an intermediate sized allele would result in an allele at high risk of more rapid expansion to a pre– and full mutation. A second high–risk haplotype for expansion in Caucasians (6–4–4/5) is infrequently seen in intermediate alleles, suggesting more rapid progression to a full mutation. The AGG interspersion pattern in normal alleles on these haplotypes is characterized by two AGGs and a middle CGG tract longer than the terminal tracts. This unusual repeat substructure may predispose to rapid expansion. In other ethnic groups different haplotypes are more often associated with expansion, e.g., 4–4–5 and 3–4–5 in African Americans [Crawford et al., 2000b]. These haplotypes may be related to the 6–4–4/5 haplotypes seen in Caucasians, as they are similarly not associated with intermediate alleles and may have progressed rapidly to a full mutation. Despite CGG expansion mutations occurring on many different haplotypes in different populations, the most common haplotype on normal chromosomes in Caucasians is 7–3–4+ and in most studies there is a negative association between this haplotype and expansion, indicating that this background may be protective against instability. Finally, approximately 15% of fullmutation chromosomes in some studies have a microsatellite haplotype that is rare in the normal population, which may suggest a mechanism in some individuals of a more general microsatellite instability that generates expansions at the FRAXA CGG repeat but also at flanking dinucleotide repeats, thus generating unusual haplotypes [Ennis et al., 2001].

The aim of this study was to locate potential *cis* modifiers of instability using a dense coverage of SNP markers in the FRAXA region.

## MATERIALS AND METHODS

### Sample

Our diagnostic facility, previous research, and a large survey of fragile X chromosomes [Youings et al., 2000] has resulted in the accumulation of over 7,000 independent samples, primarily of Caucasian origin, each genotyped for the FRAXA trinucleotide repeat and adjacent microsatellite markers. From this cohort, we selected all male samples carrying a CGG repeat tract in excess of 40 repeats for which sufficient viable DNA remained (n5285). The breakdown of the CGG repeat size distribution for these chromosomes was 144 with 41–50 repeats, 69 with 51–200 repeats, and 72 with greater than 200 repeats. For each of these 285 expanded cases, we determined their DXS548–FRAXAC1–FRAXA2 haplotype and from our male samples, selected two chromosomes with the same haplotype harboring CGG repeat sizes in the normal range (0–40) as matched controls (n=5592). The resultant panel comprised 877 unrelated chromosomes representative of the diversity of microsatellite–based haplotypes and of the range of repeat sizes within these haplotypes. Seven primate samples (1× *Pan trogolodytes*, 1×*Pan paniscus*, and 5×*Gorilla gorilla*) were also included for genotyping.

### Molecular Analysis

To enrich our genotyping effort for polymorphisms that might be associated with CGG repeat expansion, we used heteroduplex analysis for SNP discovery to compare expanded with normal alleles on a variety of microsatellite haplotype backgrounds. We used a panel of 36 females with one expansion and one normal chromosome and amplified 400–bp regions of DNA distributed at approximately 20–kb intervals over a 650–kb genomic region that included the FMR1 gene (GenBank accession number L29074.1) and 400 kb of upstream and 250 kb of downstream sequence from the FRAXA CGG repeat. The 400–bp PCR fragments formed heteroduplexes for analysis on the Transgenomic WAVE Machine (Transgenomic, Inc., Omaha, NE) and samples with shifted peaks were sequenced to confirm and characterize the variant [Brightwell et al., 2002]. SNPs with minor allele frequencies >5% (i.e., found in two or more individuals) were chosen for genotyping in our male panel. If no SNP was detected in a particular fragment, an adjacent 400–bp fragment was analyzed. Genotyping was by allele–specific PCR analyzed on ethidium bromide–stained agarose gels [Brightwell et al., 2002] (Primer sequences available in Supplementary Tables S1a and S1b; available online at http://www.interscience.wiley.com/jpages/1059-7794/suppmat). Hardy–Weinberg equilibrium could not be tested in our male hemizygous samples but duplicate samples included for quality control purposes were concordant.

The 31–kb region of highest association with expansion was screened for possible deletions and duplications by amplifying six overlapping fragments of between 2.7 and 8.5 kb by long–range PCR (sequences and locations of primers in Supplementary Table S2). Briefly, 50 ng of DNA was amplified with the Expand Long Template PCR System (Roche Diagnostics, Basel, Switzerland) following the manufacturer's protocol, using an annealing temperature of 55°C and an 8–minute elongation step in each cycle of the PCR reaction. Products were separated on 0.8% agarose gels and visualized by ethidium bromide staining. We tested only females for deletions/duplications, as we assumed they would be heterozygous and therefore make it easier to detect a novel band of different molecular weight. Pilot experiments demonstrated our ability to detect differences of about 500 bp for fragments of 6kb. All gels were scored by two independent observers and strong bands were diluted and rerun to ensure doublet bands were not obscured. The insertion/deletion analysis was performed on all female relatives of males with FRAXA expansions used in the SNP genotyping experiments, who shared an X chromosome and had sufficient DNA for analysis. There were 182 females who matched these criteria.

Sequencing templates were prepared by long–range PCR, as described for insertion/deletion testing, but using 15 overlapping fragments of between 2.5 and 5 kb (see Supplementary Table S3 for primer sequences and location). Sequencing of each template and subsequent analysis using Mutation Surveyor software (SoftGenetics, State College, PA) was outsourced to MWG Biotech (MWG–Biotech AG, Ebersberg, Germany).

### Association Analysis

The linkage disequilibrium (LD) patterns associated with SNP data were analyzed using the LDMAP program [Tapper et al., 2003, 2005]. This program calculates distances analogous to the centiMorgan scale of linkage maps where plateaus or *blocks* depict genomic regions of high LD and intervening steps characterize the magnitude of LD decay between blocks.

Association analyses were conducted using the LOCATE program [Maniatis et al., 2005]. Briefly, this method computes association *(z)* and corresponding information (K_*z*_) between each SNP and the fragile X expanded/not expanded phenotype and models the decline of association with distance using composite likelihood. The program estimates Ŝ, the location of the disease locus (or in this case associated cis effect) and computes the supporting 95% confidence interval (CI).

Other statistical analyses were performed using SAS V8.2 (SAS Institute Inc., Cary, NC)

## RESULTS

### Association Analysis

**FIGURE 1:**
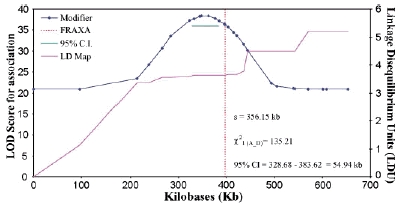
FIGURE 1. Results of the preliminary association mapping analysis and an LD map of the region created using data from all 877 individuals.The LDpattern is particularly flat in the region adjacent toFRAXA (200–400 kb), indicating very high levels of LD. Aplot of the LODscore for association shows the highest evidence for association at ∼350 kb.

Genotype data for 29 SNPs and one dichotomized microsatellite were determined for the entire sample (Table 1). SNP coverage extended approximately 650 kb, from˜400 kb proximal to the FRAXA repeat to ˜200 kb distal. By considering all chromosomes expanded at the FRAXA locus (CGG>40) as affected and all nonexpanded (CGG≤40) chromosomes as controls, we examined these data for evidence of significant association between the fragile X repeat and any possible *cis*–acting factors. Data from all 30 markers were formatted for an association analysis using the LOCATE program [Maniatis et al., 2004]. Individually, many of the SNPs showed significant association with repeat expansion but the highest single chi–squared statistic was observed for a locally ascertained SNP ss71651738 (local id WEX70) (χ^2^_1_=178.71). Composite likelihood analysis of these data indicated a point location at 356 kb on the local map with a 95% CI for association of approximately 55 kb extending from 328.68 kb to 383.62 kb (Fig. 1). The maximum LOD score identified for the likelihood curve was 38.37 indicating very strong evidence in favor of some determinant 5′ of FRAXA that predisposed to larger repeat sizes. In order to elucidate the precise nature of this putative *cis effect*, we initiated an in–depth study of the 55 kb CI indicated by the association results.

**TABLE 1:** Genotyped SNPs and Their Kilobase Locations Flanking the FMRI Gene (GenBank Accession Number L29074.1)*

		UCSC Genome Browser		Relative to	
			
Local marker ID	rs/ss number	May 2004	March 2006	FRAXA	5′
WEX54	rs555559	146301138	146403284	−397977	0
WEX32	ss71651735	146397661	146499807	−301454	96523
**DXS548**		**146509093**	**146611239**	**−19022**	207955
WEX28	rs17312728	146514865	146617011	−184250	213727
WEX83	rs236024	146539471	146641617	−159644	238333
WEX86	ss71651736	146566561	14668707	−132554	265423
WEX44	rs1868140	146584284	146686430	−114831	283146
WEX88	rs4824253	146623814	146725960	−75301	322676
WEX89	ss71651737	146632990	146735136	−66125	331852
WEX70	ss71651738	146645208	146747354	−53907	344070
WEX74	rs2121749	146645391	146747537	−53724	344253
WEX76	ss71651739	146645589	146747735	−53526	344451
rs2197711	rs2197711	146649557	146751703	−49558	348419
WEX106	rs5904647	146663426	146765572	−35689	362288
WEX82	rs5904648	146677719	146779865	−21396	376581
WEX85	rs25705	146686062	146788208	−13053	384924
**FRAXACI**		**146691923**	**146794069**	**−7192**	390785
WEX1	rs10521868	146697050	146799196	−2065	395912
WEX5	rs1805420	146697852	14679998	−1263	396714
**FRAXA**		**146699115**	**146801261**	**0**	397977
ATLI	rs4949	146704511	146806657	5396	403373
**FRAXAC2**		**146711547**	**146813693**	**12432**	410409
FMRB		146715936	146818082	16821	414798
rs25715	rs25715	146722838	146824984	23723	421700
rs25704	rs25704	146737084	146839230	37969	435946
WEX20	rs6626286	146745163	146847309	46048	444025
rs764631	rs764631	146793795	146895941	94680	492657
WEX17	rs12010481	146806449	146908595	107334	505311
WEX103	ss71651740	146839699	146941845	140584	538561
WEX52	rs5904668	146843890	146946036	144775	542752
WEX97	rs6626992	146871500	146973646	172385	570362
TTG1	ss71651742	146902096	147004242	202981	600958
WEX58	rs4588989	146910116	147012262	211001	608978
WEX10	ss71651741	146953852	147055998	254737	652714

* Microsatellites including the FRAXA repeat are presented in bold font and these variants were not used in the association analysis. Analysis of our sample at the ss71651742 (local idTTG1) locus yielded only twovariants (5 and 6); this bialleleic genotype lent itself to use in the associationmapping program and for this reason ss71651742 was treated in the same manner as SNPs.

rs, reference SNP; ss, submitted SNP

We initially hypothesized that a variant which affected stability of the triplet repeat lying up to 75 kb distal might be a relatively large deletion or insertion that would affect the chromosomal structure or perhaps remove or insert a putative stability modifier. Hence, we screened the region of most significant association for deletions and insertions. The analysis was limited by the amount and quality of the DNA available to test and the resolution of the method used. We estimated that the method would detect deletions/insertions of greater than 500 bp, and obviously not larger than the PCR products amplified in the analysis, i.e., 2.5–8.5 kb. Larger deletion/insertion testing would require molecular cytogenetic techniques, which was not feasible. We found no evidence of any deletions or insertions in the 182 females tested.

Absence of evidence indicating a chromosomal deletion to be responsible for the signal of association necessitated a more comprehensive analysis of all possible variation contained within the 55–kb interval. The cost of genomic sequencing of such a large interval in all 877 males constituting our SNP panel was prohibitive. We therefore limited this part of our analysis to those chromosomes that through empirical study have shown both the highest and lowest risk of CGG repeat expansion. A previously described classification system for FRAXA chromosomes established five main haplogroups (A–E) based on the DXS548, FRAXAC1, and FRAXAC2 microsatellites (Table 2) [Ennis et al., 2001]. Repeat size distributions, CGG repeat interspersion patterns, and ratios of normal to expanded chromosomes were found to be group–specific. Haplogroup A (7–3–4+) is the most common haplogroup found in northwest Europe and has the lowest ratio of expanded to normal FRAXA alleles. Haplogroups C (6–4–4/5 and first order derivatives) and D (2–1–3 and first order derivatives), although distinct in their interspersion patterns, FRAXA allele distribution, and possible modes of expansion [Eichler et al., 1996], have in common an inflated ratio of expanded to normal chromosomes. A total of 182 chromosomes were randomly selected from haplogroups A, C, and D in normal/expanded ratios of 27/25, 29/29, and 31/41, respectively. These samples were included with 6× duplicates and 4×water controls on 2×96–well plates for sequencing. The resultant sequencing data for all samples were aligned against the May 2004 release of Absence of evidence indicating a chromosomal deletion to be responsible for the signal of association necessitated a more comprehensive analysis of all possible variation contained within the 55–kb interval. The cost of genomic sequencing of such a large interval in all 877 males constituting our SNP panel was prohibitive. We therefore limited this part of our analysis to those chromosomes that through empirical study have shown both the highest and lowest risk of CGG repeat expansion. A previously described classification system for FRAXA chromosomes established five main haplogroups (A–E) based on the DXS548, FRAXAC1, and FRAXAC2 microsatellites (Table 2) [Ennis et al., 2001]. Repeat size distributions, CGG repeat interspersion patterns, and ratios of normal to expanded chromosomes were found to be group–specific. Haplogroup A (7–3–4+) is the most common haplogroup found in northwest Europe and has the lowest ratio of expanded to normal FRAXA alleles. Haplogroups C (6–4–4/5 and first order derivatives) and D (2–1–3 and first order derivatives), although distinct in their interspersion patterns, FRAXA allele distribution, and possible modes of expansion [Eichler et al., 1996], have in common an inflated ratio of expanded to normal chromosomes. A total of 182 chromosomes were randomly selected from haplogroups A, C, and D in normal/ expanded ratios of 27/25, 29/29, and 31/41, respectively. These samples were included with 6×duplicates and 4×water controls on 2×96–well plates for sequencing. The resultant sequencing data for all samples were aligned against the May 2004 release of the human genome reference sequence (http://genome.ucsc.edu). A total of 212 variant sites were observed, of which only seven had been previously identified in our sample. However, many of thes variants were unique to a single chromosome (n=92), and others had a minor allele frequency <5% (n=64). For the entire sample of 182 sequenced chromosomes, 56 common variants composed of SNPs (n=47), single base pair insertion/deletions (n53), and six other small insertion/deletions (1× 4–bp del, 1×3–bp ins, 2×4– bp ins, 1×5–bp ins, and 1×8–bp ins) remained. All variants were recoded into binary form, representing biallelic SNPs and presence/absence of other mutations. These data were added to our existing SNP data from across the 650–kb FRAX region and formatted again for association analysis using composite likelihood.Affection was assigned as before in the combined sample of 182 chromosomes from all three subgroups. Evidence for association between expansion status in this sample and the region although suggestive, was not formally significant (χ^2^_1_=3.13, p=0.08). We repeated the same composite likelihood analysis of association but limited the analysis to the 72 D haplogroup chromosomes alone. Despite the sizeable reduction in sample size and therefore power, we found the LOCATE analysis illustrated significant evidence of association between the D group and the region (χ^2^_1_=9.93, p=0.0016). However, the CI for association included the FRAXA repeat and we could not conclusively determine any impact other than the triplet repeat itself. The ss71651738 variant was the only SNP within the sequenced region to yield a chi–squared statistic for association>10 in both the entire sample of 182 sequenced chromosomes (n=182, χ^2^_1_=11.40) and the D haplogroup (n=72, χ^2^_1_=17.84) analyses.

**TABLE 2:** Microsatellite–Based Haplogroup Characteristics

	Microsatellite based haplogroups				
	
	A	B	C	D	E
DXS548–FRAXAC1–FRAXAC2	7–3–4+only	FOD of 7–3–4+	6–4–4/and FOD	2–1–3 and FOD	All other
Alternative nomenclature^a^	40–38–		42–36–58/60	50–42–62	
Primary and secondary modal CGG repeat number^b^	30, 20	30, 29	32, 30	29, 33	

a For further information see Chiurazzi et al[1999]

b For further information on haplogroup insterspersion patterns, etc, see Ennis et al.[2001]

FOD=first order derivatives i.e.derived from a given microsatellite pattern with a repeat size changed at one marker only

### ss71651738

**FIGURE 2:**
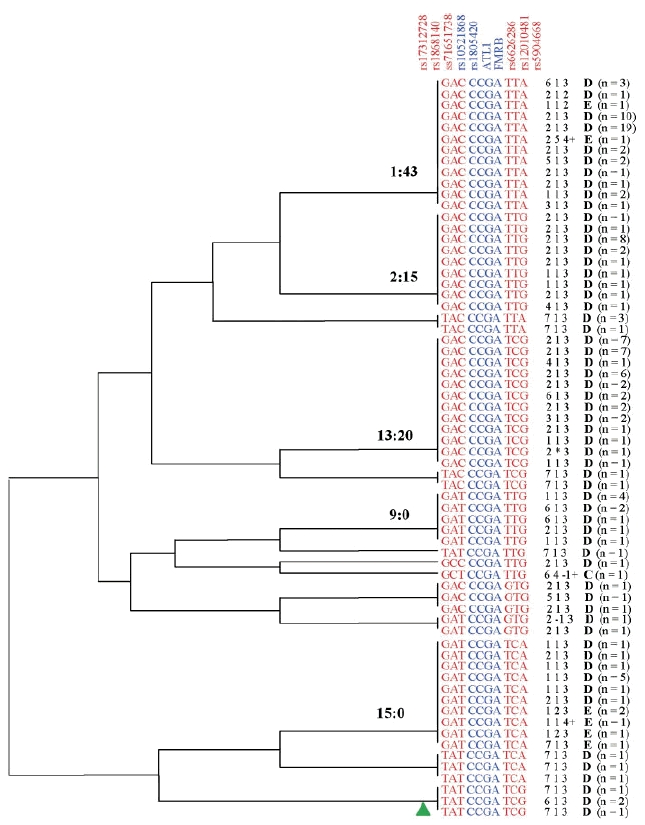
FIGURE 2. Unrooted phylogenetic tree representing the CCGA core haplogroup.The alleles for each of the four SNPs used to identify core groups are highlighted in blue (rs10521868, rs1805420, ATL1, FMRB).The six SNPs used in the subsequent stage of analysis are highlighted in red (rs17312728, rs1868140, and ss71651738 on the left–hand side; and rs6626286, rs12010481, and rs5904668 on the right–hand side).The black type that follows the SNP haplotype shows the DXS548–FRAXAC1–FRAXAC2 microsatellite haplotype followed by the corresponding microsatellite haplogroup and the number of observed instances of each haplotype (these data in black are for annotation purposes only and were not used in the phylogenetic analysis).The normal to expanded ratio of FRAXA chromosomes is shown for each branch.The branch onwhich the only observed primate haplotype co–occurs is identified by a green arrow. ^*^Denotesmissing data.

Results from a phylogenetic study conducted on a subset of the SNP data genotyped in our panel of 877 males, also distinguished ss71651738 as being of particular interest [Ennis, 2003]. In the first phase of the study, four SNPs located physically closest to FRAXA (rs10521868, rs1805420, ATL1, FMRB) were used to cluster chromosomes creating five “core haplogroups.” Microsatellite information was not used in the analysis. These SNP based haplogroups captured much of the group specific characteristics previously observed in the microsatellite based haplogroups. The “CCGA” core haplogroup was of particular interest. An unrooted phylogenetic tree of this group was created using the UPGMA option from the PHYLIP suite of software (Fig. 2) [Felsenstein, 1989]. The CCGA group contained 157 out of 159 of the D haplogroup chromosomes within our panel of 877 genotyped chromosomes. A solitary example from the C haplogroup was identified on the CCGA background and this particular chromosome was also unique in terms of its microsatellite composition. The addition of three SNPs proximal to the core SNPs (rs17312728, rs1868140, and ss71651738) and three SNPs distal (rs6626286, rs12010481, and rs5904668) generated an additional 14 subgroups.

Assays developed using human DNA gave clear results for rs10521868, rs17312728, rs1868140, and rs6626286 in all gorilla and chimpanzee samples and showed these loci to be monomorphic in this small sample resulting in a single haplotype. However, despite repeated efforts, assays for other SNPs were unsuccessful, presumably due to primer polymorphism, and these SNPs were typed by direct sequencing in Pan troglodytes. Interestingly, the single observed haplotype identified by our combined analyses of primate samples also occurred in two of our independent human samples. This haplotype is observed in the CCGA SNP group and is denoted in Fig. 2 by a green arrow.

Branches on the phylogenetic tree in Fig. 2 have strikingly dissimilar ratios of normal: expanded FRAXA chromosomes. Among the six variant SNPs in this analysis, the ss71651738C allele transmits most clearly on chromosomes with a moderate to high ratio of expanded alleles, whereas branches on which ss71651738 is found as the “T” allele have a very low incidence of FRAXA–expanded alleles and include the branch common to our primate data. The ss71651738C allele was unique to haplogroup D chromosomes. Among the complete set of haplotypes identified within haplogroups A, B, and C, we detected no examples of the C allele at the ss71651738 marker.

As ss71651738 proved of particular interest in both our association study and the phylogenetic analysis, we wanted to investigate if the C allele of this SNP appeared to cosegregate with expanded FRAXA chromosomes in other independent samples. Three international groups who were known to have examined the microsatellites adjacent to FRAXA, provided DNA from Caucasian individuals with haplogroup D chromosomes, for ss71651738 genotyping, in our laboratories. All samples designated as expanded from these international laboratories had FRAXA CGG tracts with more than 60 repeats.

Expanded chromosomes only from 11 males of Italian origin (provided by P. Chiurazzi) all showed the C allele at ss71651738. Similarly, 21 chromosomes from males with FRAXA expansions from New York were tested (provided by S. Nolin) and all but one were hemizygous for the C allele at ss71651738. A third sample (provided by S. Sherman, Atlanta, GA) contained both expanded and normal chromosomes and the results for these are shown alongside our Wessex data in Table 3. Furthermore, in the Atlanta samples, the distinction between chromosomes carrying the ss71651738C or T allele is even greater if a cutoff of 30 FRAXA repeats is used; i.e., of the 20 T alleles observed, only one had a FRAXA repeat greater than 30 (n_CGG_=32) and of the 42 with the C allele, only one had a FRAXA repeat less than 30 (n_CGG_=29) (Fisher's Exact test, p=9.14×10^−14^). There was no evidence of allelic heterogeneity between the Wessex and Atlanta ss71651738 samples (p=0.429) and we therefore also analyzed their combined evidence for the ss71651738 SNP (χ^2^_1_=70.57, p=4.44×10^−17^).

**TABLE 3:** ss71651738 Genotype Data for Haplogroup D Chromosomes From the Atlanta and Wessex populations

	CGG 0–40(%)	CGG>40(%)	p*
Atlanta			
T	20(48.8)	0	
C	21(51.2)	21(100)	
			3.4×10^−5^
Wessex			
T	38(51.4)	6(5.0)	
C	36(48.6)	114(95)	
			7.5×10^−14^

* Using Fishers exact test

## DISCUSSION

Using X chromosomes collected over decades of research and diagnostics performed on the FRAXA triplet repeat and adjacent markers, we have investigated 877 samples from males carrying both normal and expanded alleles. Positive association between fully–mutated FRAXA alleles and microsatellite haplotypes have been reported over the years for many different ethnic groups—our aim was to use less mutable SNP variants to examine the evidence for association between expanded alleles and any genetic alteration that may be acting in cis and predisposing to expansion.

A panel of 30 variants, predominantly comprised of SNPs, were identified and genotyped across a 650–kb region surrounding the FRAXA repeat. We used the LOCATE program, which employs a composite likelihood approach to examine all data simultaneously, and found a significant signal for association proximal to the CGG repeat. The ∼55–kb CI associated with this point estimate was used as the focus for more detailed analysis.

We began our in–depth analysis of this region by screening for deletions but found no evidence indicating that genomic deletions were responsible for the association signal. However, our method was limited to detecting deletions between 0.5 and 5 kb and it is possible that very small or very large anomalies could have been missed. To comprehensively screen this region for determinants of expansion, a sequencing experiment was designed. Because of financial constraints, it was not feasible to sequence this region in our entire sample and we therefore selected a subsample of 182 chromosomes from haplogroups A, C, and D. Previous microsatellite studies suggested these haplogroups represented both the most and least stable chromosomes with regards to FRAXA repeat size. We hypothesized that inclusion of chromosomes from either end of the stability spectrum would increase the probability of identifying a causal cis–acting variant. Sequence analysis revealed 43% of genetic variants were unique to a single chromosome and a further 30% were very rare (MAF<5%). No single detected variant cosegregated with the expanded alleles alone and association analyses of all data on either the sample of A, C, and D haplogroups combined or the D haplogroup alone could not determine any significant effect that excluded the FRAXA CGG repeat itself.

The ss71651738 SNP was consistently significantly associated with expanded chromosomes in both our preliminary analysis of 877 males and subsequent sequence analysis of a subsample of 182. Partitioning the data into the microsatellite–based subgroups indicated that this association was entirely derived from the D subgroup. This result concurs with the findings of an alternative analytical approach, in which a study examining the phylogenetics of X chromosome SNP haplotypes revealed that the C allele of ss71651738 SNP was almost exclusive to an SNP–based “CCGA” core haplogroup which incorporated almost all microsatellite haplogroup D chromosomes. Furthermore, within this SNP haplogroup, the C allele of ss71651738 accurately identified tree branches with a very high proportion of fragile X expansions. Interestingly, the single SNP–based haplotype we observed in primates also carried the “CCGA” SNP core common to most of our D group data. At ss71651738 the primate sample bears the T allele, which in the human data appears to distinguish chromosomes at reduced risk of expansion.

To exclude the possibility that our unusual findings with regard to this SNP were unique to our sample of males from Southeast England, we genotyped this SNP in a number of normal and expanded haplogroup D X chromosomes from three independent Caucasian samples. Despite limited sample sizes in these new data, we observed no evidence for genetic heterogeneity between samples and found the ss71651738 allele distribution in the normal and expanded groups to be very similar to that observed in our Wessex data. In fact, within the sample provided by our colleagues in Atlanta, the C allele almost perfectly partitioned the FRAXA CGG alleles above 30 triplet repeats.

The mechanism of triplet repeat instability is unknown and may occur during DNA replication, DNA repair, or by gene conversion or recombination [Cleary and Pearson, 2005]. *Cis* elements that might affect the instability mechanism include sequence elements within and without the repeat, CpG methylation, nucleosome and replication origin positioning, CpG density, and transcription levels [Pearson et al., 2005]. Two recent works report origins of replication for FMR1, both within 600 bp proximal to the CGG repeat [Brylawski et al., 2007; Gray et al., 2007]. The origin is used in both normal and expanded/methylated repeats. The CGG repeat would be replicated on the lagging strand template, which would predict that contractions of CGGs would be more common than expansions. In model systems expansions have been shown to be more common when the template is on Okazaki fragments and in the case of FMR1 would therefore be generated following replication from an origin distal to the CGG repeat [Cleary et al., 2002]. The region we have identified as a modifier of stability is proximal to the repeat and our 95% CI does not include the proximal replication origin. It is therefore unlikely that the candidate modifier we have located is an origin of replication.

The region is conserved in other species, but there are no known genes within the 54.9–kb region. There are, however, two ESTs: a 603–;bp sequence (CD721808) and a sequence of over 450 kb (AL698651). CD721808 was isolated from a lacrimal gland cDNA library [Ozyildirim et al., 2005]. The AL698651 sequence aligns to six other sites on different chromosomes with approximately 97% homology. The most significantly associated SNP in our analysis was ss71651738 and this SNP lies within a 340–bp medium reiterative element 1B (MER1B) sequence (location 146,645,059–146,645,398). MER1B sequences are often found in the 50 regions of genes and may contain Alu sequences, but are not thought to have arisen by retrotransposition [Jurka, 1990]. These elements have been associated with genome instability, in particular in the dystrophin gene. Intron 7 of dystrophin contains several repetitive elements, including MERs, and this intron has expanded approximately 44–fold over 400 million years [McNaughton et al., 1997]. A novel promoter for dystrophin was identified 500 kb upstream of the previously reported promoter and it has a sequence similar to a MER element [Nishio et al., 1994]. These elements have also been associated with tightly bound DNA–protein complexes in eukaryotic chromatin [Avramova et al., 1994].

It is conceivable that DNA–protein interaction at the MER1b site, including ss71651738, affects chromatin structure and could influence replication of the CGG repeat. A recent study of chromatin conformation in a 170–kb region encompassing the FMR1 gene revealed that expressed vs. repressed states of the FMR1 gene exhibit differential chromatin interaction profiles –Gheldof et al., 2006]. The most striking differences were observed in a 50–kb segment centered on the FMR1 promoter. No notable differences were observed in the region sequenced in the current study.

One of the most comprehensive studies of the fragile X haplotypes on an ethnic group other than Caucasians was that by Crawford et al. [2000b] on an African American population. The distribution of microsatellite–based haplotypes observed in this population was predictably distinct from that observed in Caucasians. Group D haplotypes accounted for only 87% of mutated chromosomes in the African American population but over 32% in a Caucasian sample from the same study. Various bottlenecks (caused by slavery, war, ice ages, famine, and plague) each time followed by unknown founder effects, makes extrapolation of precise evolutionary history fraught with error. The difficulty is further compounded by research that indicated mutable microsatellites to be the markers of choice.

ATL1 represents the first SNP marker to have been thoroughly investigated for association with expanded FRAXA repeats [Gunter et al., 1998]. The strong association observed by Gunter et al. [1998] was later shown by studies in non–Caucasian populations to be a likely founder effect in Caucasians rather than causal. The same studies indicated the ATL1 G allele was likely to be ancestral [Crawford et al., 2000a; Kunst et al., 1996]. Our results are consistent with these findings. Using SNPs tightly linked to the FRAXA locus (rather than more remote and mutable microsatellites), the ATL1 G allele is found at position 3 in the “CCGA” SNP–based group. Our primate samples belong to this diverse group. Although the ATL1 A allele has inflated frequency in Caucasians compared to African Americans, this is substantially influenced by allele A co–occurring with the very common 7—3—4+haplotype (microsatellite haplogroup A). It is plausible that the ATL1 A allele is recently derived but has hitchhiked to appreciable frequency in Caucasians on the “protective” 7—3—4+haplotype.

Our findings are suggestive of an ancestral origin of the CCGA SNP–based haplogroup and therefore of the microsatellite–based D group of which it is mainly composed. The ss71651738 SNP occurs 3′ of the SNPs defining the CCGA group and both its C and T alleles are found on this background. The T allele occurs on haplotypes with normal–sized CGG repeats and on our primate haplotype. The C allele appears to transmit with a disproportionate number of expanded chromosomes and may be of clinical importance in assigning high/low expansion risk.

Our LD map of the FRAX region exhibits extensive allelic association similar to the findings of Mathews et al.[2001]. On close inspection, there appears to be evidence of a small increment on the LD scale indicative of historical recombination coincident with the FRAXA CGG repeat itself. The region immediately proximal to the FRAXA triplet is uniformly flat on the LD scale, extends for more than 50 kb and includes the ss71651738 SNP. Although somewhat speculative: 1) absence of evidence for historical recombination in the sequence between ss71651738 and FRAXA; 2) relative diversity of haplotype backgrounds on which the ss71651738C alleles occurs; and 3) absence of the C allele on non haplogroup D chromosomes; suggests that the C allele of the ss71651738 SNP arose on chromosome(s) from the D haplogroup soon after the recombination events that produced haplogroups A, B, and C. The various SNP–based haplotypes found on the D background, however, are more likely to have arisen from recombination events. A *relatively* large increment on the LD scale between FMRB and rs6626286 suggests some point in the intervening sequence to be commonly involved in crossover events. Similar, but smaller steps between LD blocks occur either side of the rs1868140 SNP. The pattern of LD observed in this study is consistent with that produced using data from the HapMap project [Tapper et al., 2005].

This is the first study that has attempted to locate and identify a potential modifier of CGG repeat stability in cis with the *FMR1* gene. Composite likelihood analysis of SNP genotypes from 877 expanded and control X chromosomes covering 650 kb flanking the CGG gave a point estimate very close to the ss71651738 SNP, which when examined singularly, was the most significantly associated SNP (χ^2^_1_=178.71). The 95% CI for this point estimate covered a ∼55–kb region proximal to the CGG repeat and we conducted detailed analysis of this signal in a smaller sample. We failed to identify any linked insertions, deletions, or other genomic polymorphisms that were more significantly associated with expansion status than the ss71651738 SNP. We further demonstrated that the association was predominantly due to expansions on one particular branch of the phylogenetic tree. These data suggest that ss71651738 is within the functional modifier locus affecting instability of FRAXA, and we propose a possible role for the MER1b element in which it lies. Further investigation of this SNP in functional studies and samples of diverse ethnicity will help to establish the etiology underlying our observations.
