# Supplementary material for: Closely linked cis-acting modifier of expansion of the CGG repeat in high risk FMR1 haplotypes
Source: Hum Mutat. 2007 Aug 2;28(12):1216–24. doi: 10.1002/humu.20600 (PMC2683060; doi:10.1002/humu.20600)
Supplement: Supplementary file 1 [file humu0028-1216-SD1.pdf]

Supplementary Tables S1-S3

Supplementary Table S1a. Generic primer sequences for locally typed SNPs

Local SNP ID	rs number	Forward Primer	Reverse Primer
WEX54	rs555559	ATTCATTTCTGCCATCACAGAGCTTGTT	TGGAATATAAATTTAACAGATTATTTGTTG
WEX32	ss71651735	ACCATAGCAGTGGCATAAAAACAGACATATA	TGCTTCTGAGGTCTTTTTCTAAAAATTCTTG
WEX28	rs17312728	TTAATAAGTGATCAATTGATGACTGC	AGCAATATCTTAAATTAGATACCCTTACTG
WEX83	rs236024	TCAAAATCACAGATTAGACTTCCCTTGAAG	ACACGACTTAGCTTGAGGTTCTCTGACTAG
WEX86	ss71651736	ACCTGTCTCTTCTCCATCAAATACAAACAG	TCCAAGAAAAAGTGGTTAAGAGACTAGGGT
WEX44	rs1868140	AAATTTTCTCTGCTCTGAGTTTACGC	GGAGATCAAAAAGGAAATAGTAACTAGG
WEX88	rs4824253	TTTCTACTTCTCTCTCATACTTCGGTACCCA	GATCAGATAGATAAAACCGCATTCCAAAAC
WEX89	ss71651737	CAAATTCTGACCCAAACACCATTTATCATA	GCAACATAGTGAGACCCCATCTCTAAAAAT
WEX70	ss71651738	GGA GTT ATT GTC CTA TAC AGC AGT TGT CCC	GAT GGG AGA TGA TTT TGG GAT AAA ATT
WEX74	rs2121749	GCAACTTCAGGCTTGCTACC	GGCGTGTAAACCCTATCGTG
WEX76	ss71651739	GCAACTTCAGGCTTGCTACC	GGCGTGTAAACCCTATCGTG
NA	rs2197711	GAGCAGCAAATATGCAGAGCTAATCTACTC	TAATTTTGTTAATGATGGTCTGCTGGTTCT
WEX106	rs5904647	TCTGTGTCTCCTGAATTGCAATTCCTAAGA	GAGGTTCAGGGACACAAGAGGACTCAC
WEX82	rs5904648	AGACAAGTCAAACTATGAGAGGATAT	TTCCAAAGTGTTTATATGTGTACATAC
WEX85	rs25705	TATAGTCAAATGTAACCAGATCAATAAACA	TAATATTGAAGCAGAGAAATATTTCTAAGC
WEX1	rs10521868	TTCTGGCATACTCGGTAGCA	TCATCTTTCTCGATCCCCAC
WEX5	rs1805420	CTTCTCCACCCCAGACTAGGTCATGT	CAGACACTGTGCTAAGTGCTAACGAGAAAT
ATL1	rs4949	ACCCTGATGAAGAACTTGTATCTCT	GAAATTACACACATAGGTGGCACT
FMRB		GTGATAGAATATGCAGCATGTGATGCAAC	CGGAGAAATCGTTTATAGTTTGTGACTTATGACA
NA	rs25715	AAGCAGTCATTTTCCAGTAATGTATA	TATACATACATCCAAAAGGAATACAAAT
NA	rs25704	TTGGAAAAGACTAAGATCGGTTAACAAATCAT	CAAAAAACAAAAGAAAAAAAGAAAAAAAAGGTG
WEX20	rs6626286	CCTCAACTATAGTGCAGTTTCAAGTACGTTTCAACC	TGAGACCTCATTTCTAAAACCAAACAAAAAAGAGG
NA	rs764631	TGCCATTAAATGTTTGCTTTTCTTCTTTGTCT	CAAATATGAACTTATGCCACTGCGATCTGT
WEX17	rs12010481	ATTTAACTCCAGAATTTCTGCCTGATT	GCCTCTGATGGTAATCCAGAGAATTAT
WEX103	ss71651740	GAA AAA AAT GTG AAT CAG TCA CCA CTG GAA	GGA AAT TAG CCT GAG GCT TAC CTT TCA TTG
WEX52	rs5904668	AGCTCAGATCCTAGTAGGTTTCTTGAGCTAA	TACTCCCCTGTAACTCTAAGCTTTCTGATG
WEX97	rs6626992	GCAGAAGGCCAGTTAAGAAGTGAGTCAA	ACTAAAAATTCACAGCAGTTCCAGGGC
TTG1	ss71651742	GAGTATTGTAAATAGAAAGGGTTG	CTGTCTCCTTCTCTGGTTCTG
WEX58	rs4588989	GACATTGGACTGCCTGGTATAACAAACAA	TATGAACTCTTGGACGTTGATTGCCATCAA
WEX10	ss71651741	GATACATATTTGTTGTTGTTGGAAACACTG	AACACTGTAAGCGATGGAGAATTTAACATA

Supplementary Table S1b. Allele specific primer sequences for locally typed SNPs

Local SNP ID	rs/ss number	Allele specific forward	Allele specific reverse
WEX54	rs555559	AAAGATCTCATGTTTTCACCTACCAAGT	ATTTAACAAGGTAACACATATTTACAGCCC
WEX32	ss71651735	CCAAGAACACATATTGAGGTAAGGACAATC	CAGTTATTCCAGCACCATTTATTGAACAC
WEX28	rs17312728	GAGTATGTATGTGAACTATTTTTAATCCCAT	GAGGAGTATGGGAATAGTTTACATTC
WEX83	rs236024	CCAAAAGAAAAATATGTTTTTCTCCACGA	TTTGTCTCTCTCTTCATTTCTTTGGGC
WEX86	ss71651736	CAAGTTCACCCTACCCAAAAGTAAAGGT	ACATTGAGGAATAAGAGTAAAAAAAAGGCAT
WEX44	rs1868140	CAGAGAATAGTTTCAGTTTCTCAGTTTAAACTC	CCTAACAGTATAGACCATGATGGAAACATAT
WEX88	rs4824253	TCAGCTCAGTAAGAGGCGGGTATTTTA	GCTGTTTCTCCATCTTTTCCAATACCTTATC
WEX89	ss71651737	GAGCTTACTTCATTTTGGTGGAGGGTC	CCACTTACAGAGGCCTTCAAAAACTAGC
WEX70	ss71651738	CTG TCA GAT CAT CAG GCA TTA AAT TTT AAT	AGT TCA CGA TAG GGT TTA CAC GCA TG
WEX74	rs2121749	GCAACTTCAGGCTTGCTACC	GGCGTGTAAACCCTATCGTG
WEX76	ss71651739	AGACTGCGAGATGGGAGAAG	GGTAGAGACGCAGAGCCAAG
NA	rs2197711	TTTGATTATTTTATTGGCTGTATTCCTAGCAA	CAAAAGTTTATACACTTTAACAAAGTGATCCGAC
WEX106	rs5904647	CTTCTTGGTTGACATTAAATGGTGTCAGAATATAA	CAGGGGAAAGTTGCGTTGTACCCAAC
WEX82	rs5904648	TTCCTCTGATTATTAATTTATTAATGGG	TATAACCATGTAAAAAGATCTTCAAGT
WEX85	rs25705	CTCCTGGAAAAATTCTAAACACTATTATC	GTCTTTCAGAAGTGTGACTCTGTTTATAA
WEX1	rs10521868	CATTATCTGTGTTAAATTATCAAGGATCAA	AAGCTGCAATATGTTCGATGG
WEX5	rs1805420	CATACCCCTTATCACAGCTGCAACTAGTC	GTGGACATAAATCAAATTGTCAGACAAGTAATTC
ATL1	rs4949	TGTACATTTTCCAAATGCAAAGA	TTTATAAGAGACACAGAATCATAAATCC
FMRB		TCAGCATTTCTTGTGTATCAACTTACATTTCC	AGCTGGATGTGCCAGAAGACTTACCA
NA	rs25715	TTAATAGATGTTACTGAAAAATACTTACAT	AACCACAATGAAAAAGCTTTAAACAAG
NA	rs25704	TTCCATGTATGCATAATAATCCTGCAAAGT	TTTTACGGCTCTGTACAGATTCAGTGGTAC
WEX20	rs6626286	GTGCATACACAGTGGTGGATCCAGAAGGCT	GCATAATTGACTGACAGCCCCAGATGCAC
NA	rs764631	CTTGCAATCTGAGGGAAAATCAGGTAGT	TAAGATCTTGCAGCTCATTACAAGGCTCTG
WEX17	rs12010481	TTGAATTCTTTGTTTGAAAGATAATATGTCTC	CACCAGAAACTAATCCTGGAGAAACTA
WEX103	ss71651740	ACT TCA TTG AGA CAC ATT ACC TGA ATG CGT	ACC CAA TTC TTT GAT GGC TTT CAC ATG
WEX52	rs5904668	CAATCTCATTAATAAAGACAAATAGGAAGATGCAT	TGAAAAAAGCACTTTTAAACATTAACCCG
WEX97	rs6626992	AACCCTGAACATAATGCATCCTTGCA	TGATGTTCAGAATGATAACTTGGAGTCCTC
TTG1	ss71651742		
WEX58	rs4588989	GTCCTCCCTCAGCCGTGTTGGACATA	TCAAGTGAAGAGGGAAAATAAGACCACCGC
WEX10	ss71651741	TACTTGGTGCAAGGAAAATATTCAATCTTT	CTCATTTGCATACTGCTGACTTCTCG

Supplementary Table S2. Primers for fragments used in insertion/deletion analysis, with locations from UCSC July03

Fragment	Forward primer	Reverse primer	Start position	End position	Product size (bp)
1	TGTGGTTTGAAAAGGGCAAATGTTC	TCCAACTTTTGGGAAAACAAAGGG	145,594,916	145,599,508	4,593
2	TGGCTTGTTTATTAAGCCCACATCT	ACCATTTAGGCTCCCAACACACAGC	145,598,356	145,604,913	6,557
3	AAAGCCTTTTGCCAGGATGGATTTC	GCTGGAATGGTACCCTCTTTCACTTC	145,604,462	145,609,465	5,003
4	TCCTCTGGTGCTTCTCTCTCAGCAA	TGGAATCCACCTTTGTGAAACTGACA	145,609,045	145,615,145	6,100
5	TGTCAGTTTCACAAAGGTGGATTCCAAT	CCCACACAAACTCATTGTTTGCCAAT	145,615,089	145,623,639	8,550
6	GGCAAACAATGAGTTTGTGTGGGACT	AAACAGAGGCATGCACAATTCCAGA	145,623,629	145,626,205	2,576

Supplementary Table S3. Primers for fragments used in sequence analysis, with locations from UCSC July03

Fragment	Forward primer	Reverse primer	Start position	End position	Product size (bp)
A	ATGGTTGTGGAGGAGAGCAGGAAAA	TTGGGTCAGAATTTGGAAGCGTAGA	145,592,437	145,595,050	2,614
B	TGTGGTTTGAAAAGGGCAAATGTTC	TCCAACTTTTGGGAAAACAAAGGG	145,594,916	145,599,508	4,593
C	TGGCTTGTTTATTAAGCCCACATCT	ACCATTTAGGCTCCCAACACACAGC	145,598,356	145,604,913	6,557
D	AAAGCCTTTTGCCAGGATGGATTTC	GCTGGAATGGTACCCTCTTTCACTTC	145,604,462	145,609,465	5,003
E	TCCTCTGGTGCTTCTCTCTCAGCAA	CCCTTCCAGAGGTATCCATGTCTGC	145,609,045	145,612,491	3,449
F	CCACCACCCACTGATTCTAATGTGC	TGGAATCCACCTTTGTGAAACTGACA	145,612,034	145,615,145	3,111
G	TGTCAGTTTCACAAAGGTGGATTCCAATA	TGTGCATTCTGCAGCTCTTAGGTGA	145,615,089	145,619,811	4,694
H	GAGAGAGAGATGGACCCCTATGCAA	CCCACACAAACTCATTGTTTGCCAAT	145,619,632	145,623,639	4,037
I	GGCAAACAATGAGTTTGTGTGGGACT	AAACAGAGGCATGCACAATTCCAGA	145,623,629	145,626,205	2,576
J	TCTGGAATTGTGCATGCCTCTGTTT	GAGCCACAGCATATGGCTTCATGTT	145,626,196	145,630,517	4,322
K	GTCATTTGTCCTGGGCTTTGCATTT	GTTGCAATTTGGGAGACACGGATTT	145,630,289	145,634,491	4,227
L	GAAACAGGCGTTTCTACCTCCAAGG	CCCTACAGGCCAGGAGAGAGTGTCA	145,634,021	145,637,616	3,597
M	CTCCTGGCCTGTAGGGTTTTCACTG	GAAAACTATTCAGAAAGCAACGGGAGT	145,637,601	145,640,802	3,228
N	CCTTCTCCCATTTTGGGGGTTAAAA	GAGTCTGACTGCAGGCACAAGGAAA	145,639,407	145,643,877	4,472
O	GTGCCTGCAGTCAGACTCTTTGAGC	GACAACGTGCTAGCAGCCCTCATTT	145,643,860	145,646,702	2,867
